# Quinoa as a naturally stress-resistant crop: current status and future promises

**DOI:** 10.1007/s44154-025-00283-0

**Published:** 2026-02-09

**Authors:** Heng Zhang, Guojun Feng, Yaozu Feng

**Affiliations:** 1 Department of Genetics and Developmental Science, School of Life Sciences and Biotechnology, Shanghai Jiao Tong University, Shanghai, China; 2 Institute of Crop, Xinjiang Academy of Agricultural Sciences, Urumuqi, China; 3 Institute of Microbiology, Xinjiang Academy of Agricultural Sciences, Urumuqi, China

**Keywords:** Halophyte, Salinity resistance, Drought resistance, Preharvest sprouting, Heat stress

## Abstract

Quinoa (*Chenopodium quinoa* Willd.), a semi-domesticated halophyte originating in the Andean region, has emerged as a promising crop for exploiting marginal lands, valued for its exceptional nutritional profile and remarkable resilience to high salinity and drought. This review analyzes the current status and future potential of quinoa as a model halophytic crop. We begin by examining the physiological mechanisms that enable quinoa to thrive in marginal environments, which have been the subject of extensive study. Thanks to the advancement in high-throughput sequencing technology, genomic resources – including the recent development of high-quality reference genomes and a *Chenopodium* pangenome – are rapidly expanding. Sequence-based genetic mapping techniques hold the promise to dissect the molecular basis of complex traits in combination with the utility of functional genomics tools such as virus-induced gene silencing (VIGS) and stable genetic transformation. Ultimately, the application of modern breeding technologies, such as phenomics, genomic selection (GS), and CRISPR/Cas, will expedite the development of locally adapted, climate-resilient quinoa cultivars worldwide.

## Introduction

Quinoa (*Chenopodium quinoa* Willd.) is a dicotyledonous annual herbaceous plant belonging to the Amaranthaceae family that originated in the Andean region of South America. While it was once a neglected crop, it has recently gained global recognition as a “super food” or “golden grain” due to its exceptional nutritional profile (Alandia et al. 2020; Bazile et al. 2016). Quinoa is a pseudo-cereal, meaning it is consumed as a grain but is botanically distinct from grasses. One of its most significant nutritional attributes is that it is a complete protein source; it contains all essential amino acids, including lysine, methionine, and threonine, which are typically scarce in true cereals (Abdelshafy et al. 2024; Ren et al. 2023). The protein content of quinoa seeds ranges from 12 to 23%, which is comparable to that of milk and superior to that of wheat, maize, and rice (Abdelshafy et al. 2024). Furthermore, quinoa is naturally gluten-free, making it a viable and nutritious alternative for individuals with celiac disease or wheat allergies (Ren et al. 2023).

Agronomically, quinoa is a highly versatile crop utilized not only for its grain but also as a vegetable and for animal feed (FAO 2013). Quinoa greens, which include sprouts, microgreens, and leaves, are emerging as novel plant foods rich in bioactive compounds (Huang et al. 2025). The leaves are exceptionally nutritious, with a protein content as high as 37.7% in dried leaves – exceeding that of spinach – and are rich in vitamins A and C, as well as soluble dietary fibers (Gomez et al. 2024; Villacres et al. 2022). Beyond human consumption, the crop serves as a valuable resource for livestock; the whole plant can be used as green fodder or as silage, and harvest residues, such as bran and stalks, are utilized as animal feed (FAO 2013).

Perhaps most importantly for modern agriculture, quinoa is a facultative halophyte. Among crops, it has the unique ability to complete its life cycle in high salt concentrations, tolerating up to 400 mM NaCl (equivalent to 40 dS/m). Yet, it can also grow optimally in low-to-moderate salinity conditions (Santos et al. 2016). Because of this trait, quinoa is increasingly viewed as a model crop for understanding salt-tolerance mechanisms in halophytes (Morton et al. 2019). It serves as a “blueprint” for identifying key traits conferring salinity tolerance, which could potentially be incorporated into major staple crops to enhance their resilience (Zhang et al. 2018).

The global food system is currently facing unprecedented pressure due to a rapidly growing population, projected to reach nearly 10 billion by 2050, combined with the degrading effects of climate change (Afzal et al. 2023; Godfray et al. 2010). One of the most critical challenges is global salinization; approximately 10.7% of the world’s land is affected by salinity (FAO 2024). Abiotic stresses, including drought, salinity, and extreme temperatures, are the leading causes of yield loss worldwide, potentially decreasing crop productivity by over 50% (Zurbriggen et al. 2010). In the Middle East and North Africa (MENA) and Central Asia regions, water scarcity and soil salinity are already major constraints to agricultural production (Choukr-Allah et al. 2016).

To address these challenges, there is an urgent need for climate-resilient crops that can maintain productivity in marginal environments (Zhang et al. 2018). Quinoa exhibits exceptional adaptability to diverse agro-ecological zones, capable of growing from sea level to altitudes of 4000 m, and tolerating a wide range of stress factors, including frost, drought, and high soil salinity. Due to this resilience, quinoa cultivation has expanded rapidly from only eight countries in 1980 to over 120 countries in recent years (Alandia et al. 2020).

This review intends to provide a comprehensive analysis of quinoa’s potential to contribute to sustainable agriculture in a changing climate. It begins by examining the physiological mechanisms that allow quinoa to thrive in harsh conditions, such as the use of Epidermal Bladder Cells (EBCs) for salt sequestration and osmotic adjustment. The review then details the molecular genetics and genetic architecture of this allotetraploid species, highlighting the recent availability of high-quality reference genomes and the identification of candidate genes for stress tolerance. Furthermore, it explores the transition from traditional selection to modern precision breeding, including the use of gene-editing technologies like CRISPR/Cas to improve traits such as yield and saponin content. Finally, the discussion concludes with future perspectives on strategies for the sustainable expansion of quinoa into marginal lands to enhance global food and nutritional security.

## Mechanisms of abiotic stress resistance

### Salinity resistance and ion homeostasis

Quinoa belongs to the Amaranthaceae family, a lineage noted for containing a high proportion of halophytic species (Flowers et al. 2010). While most crops, including major cereals like wheat and rice, are glycophytes that suffer significant yield losses at low salinity levels, quinoa exhibits remarkable resilience. It can complete its life cycle under salinity levels as high as those found in seawater (approximately 40–50 dS m or 500 mM NaCl). Quinoa growth is often optimal under moderate salinity concentrations ranging from 100 to 200 mM NaCl, suggesting an inherent requirement for salt to maximize biomass accumulation. For example, in field trials in the United Arab Emirates, quinoa produced seed yields between 0.7 and 1.05 t/ha using irrigation water with high salinity (14–15 dS/m), which is comparable to yields in non-saline traditional growing areas (Choukr-Allah et al. 2016). However, there is a threshold above which productivity starts declining. One study identified a soil salinity threshold of approximately 12 dS/m, above which seed yield declined rapidly (Chaganti and Ganjegunte 2024). At extreme salinity levels (e.g., 400–500 mM NaCl), shoot height and biomass are significantly reduced, although plants often remain viable (Hariadi et al. 2011; Roman et al. 2020). Germination is a relatively sensitive stage to high salinity, which can inhibit germination and cause asynchronous seedling establishment (Adolf et al. 2013).

High salinity imposes both osmotic and ionic stress on the plant. The primary response involves a reduction in soil water potential, which limits water uptake and can lead to stomatal closure, thereby reducing photosynthetic capacity (Hedrich and Shabala 2018). There is substantial genotypic variability regarding salt tolerance among quinoa ecotypes; accessions from the Bolivian Salares (salt flats) are traditionally considered the most tolerant, although recent screenings indicate that coastal and lowland varieties also possess significant, sometimes superior, tolerance mechanisms (Kiani-Pouya et al. 2019).

A distinguishing anatomical feature of quinoa is the presence of epidermal bladder cells (EBCs) covering the aerial surfaces of leaves, stems, and inflorescences (Dassanayake and Larkin 2017; Shabala et al. 2014). These specialized, balloon-like cells can have a volume up to 1,000 times larger than regular epidermal cells, making them potential reservoirs for water and metabolites. Historically, EBCs have been regarded as a type of salt gland and serve as “salt dumps”, facilitating the external sequestration of toxic ions (Na^+^ and Cl^−^) away from the metabolically active mesophyll cells to prevent ion toxicity (Dassanayake and Larkin 2017; Shabala et al. 2014).

Evidence supporting the sequestration hypothesis includes the mechanical removal of EBCs (brushing) that was shown to result in a salt-sensitive phenotype and increased Na^+^ accumulation in the leaf lamina (Kiani-Pouya et al. 2017). The essential role of EBC in salt resistance has also been reported in related halophytic species such as *Mesembryanthemum crystallinum* (ice plant) and *Atriplex canescens* (Agarie et al. 2007; Wang et al. 2025a). Transcriptomic analyses of EBCs reveal high expression levels of specific ion transporters (Bohm et al. 2018; Zou et al. 2017). Key transporters identified include the sodium-selective channel *CqHKT1.2* and the anion transporter *CqClC-c*, which are proposed to mediate the loading of Na^+^ and Cl^−^, respectively, into the EBC vacuoles (Bohm et al. 2018). The transport of these ions is believed to be energized by H^+^-ATPases located on the plasma and vacuolar membranes, which generate the necessary proton motive force. Furthermore, stalk cells, which connect the EBCs to the epidermis, appear to act as “traffic controllers,” mediating polar ion transport through specific channels to direct toxic ions into the bladder (Bazihizina et al. 2022).

However, the role of EBCs in salt sequestration has recently been challenged. Mutant quinoa lines that completely lack EBCs (*ebcf*) have shown no loss of salt tolerance compared to wild-type plants (Moog et al. 2022). Instead, it was postulated that accumulation of oxalic acid in the EBCs is required to deter arthropod herbivores (Moog et al. 2023). Interestingly, multiple studies indicate that EBCs accumulate potassium (K^+^) in quantities exceeding Na^+^, even under saline conditions (Moog et al. 2022; Palacios, 2024) #8598}. A recent study also reported the presence of EBC in some spinach varieties, and potassium was identified as the main ion in these EBCs (Vieira et al. 2025). By knocking down a WD40 gene, EBC density on leaves was reduced, accompanied by elevated water loss rate, suggesting a role of EBC in water retention (Kobayashi and Fujita 2024). A recent study showed that mechanical removal of stem EBCs, but not leaf EBCs, resulted in reduced transpiration and growth (Miranda-Apodaca et al. 2025). These findings suggest an evolutionarily conserved function of the EBC in water management.

To maintain turgor pressure and water uptake in saline soils, quinoa requires efficient osmotic adjustment. Unlike glycophytes that rely heavily on the de novo synthesis of energetically expensive organic osmolytes, quinoa preferentially utilizes inorganic ions (Na^+^, K^+^, and Cl^−^) as "cheap" osmotica (Adolf et al. 2013; Flowers and Colmer 2008). It is estimated that 80–95% of the osmotic adjustment in quinoa leaves is achieved through the accumulation of these inorganic ions (Hariadi et al. 2011). This strategy requires the efficient sequestration of toxic ions into vacuoles to protect the cytosol, a process mediated by tonoplast antiporters (*NHX*) and driven by proton pumps (Adolf et al. 2013).

Crucial to this tolerance is the retention of potassium (K^+^) in the cytosol. High salinity typically induces potassium leakage due to membrane depolarization and reactive oxygen species (ROS)-activated channels; however, tolerant quinoa genotypes exhibit a superior ability to retain potassium in root and leaf tissues, maintaining a high cytosolic K^+^/Na^+^ ratio essential for enzymatic function (Rasouli et al. 2021).

While inorganic ions provide the bulk of osmotic potential, organic compatible solutes play vital roles in osmoprotection and cytosolic balance: (1) Proline: Proline levels increase significantly in quinoa under salt stress (Ruffino et al. 2010). While its contribution to total osmotic potential may be low compared to ions, it functions crucially as a ROS scavenger, a stabilizer of subcellular structures, and a buffering agent for cellular redox status (Mansour and Ali 2017). Transporters such as *CqProT* have been identified in EBCs, suggesting the transport of proline from the leaf to the bladder to balance the osmotic pressure created by salt accumulation (Bohm et al. 2018). (2) Betalains: These nitrogen-containing pigments, found in the EBCs and stems of colored quinoa varieties, possess strong antioxidant properties (Imamura et al. 2018; Polturak and Aharoni 2018). They serve as ROS scavengers, protecting the photosynthetic machinery from oxidative damage induced by salinity and high radiation. (3) Soluble Sugars: The accumulation of sugars such as sucrose and glucose is observed in response to salinity, contributing to osmotic adjustment and serving as signaling molecules and energy sources for stress adaptation mechanisms (Prado et al. 2000).

Transcriptome analyses in quinoa under salt stress identified homologs of salt-responsive genes in glycophytes, such as ABA-related genes, DREB2A, and dehydrins (Burrieza et al. 2012; Ruiz et al. 2017). Virus-induced gene silencing of *CqHKT1* and *CqSOS1* also confirmed that they contribute to sodium exclusion under saline conditions (Kobayashi et al. 2025). These results indicate that conserved components are involved in the salt stress response of both glycophytes and halophytes, but the mechanisms that confer salt stress resistance remain to be revealed.

Together, these physiological adaptations – efficient ion sequestration (whether internal or external), robust retention, and the accumulation of osmoprotectants – allow quinoa to maintain water status and metabolic activity in highly saline environments.

### Resistance to drought and low temperature

Partly due to its excellent ability in osmotic regulation, quinoa also exhibits remarkable resilience to drought and low temperatures. The crop can successfully grow under severe water stress and low precipitation regimes (e.g., < 200 mm), utilizing a variety of morphological, physiological, and biochemical mechanisms to maintain productivity (Abbas et al. 2023).

A primary mechanism of drought tolerance in quinoa is its high water use efficiency (WUE), which is regulated largely through stomatal control and root system dynamics. Quinoa is frequently characterized as an “isohydric” species, meaning it rapidly closes stomata in response to water deficits to maintain leaf water potential and relative water content (Hinojosa et al. 2018; Killi and Haworth 2017). The stomatal closure is mediated by chemical signaling, specifically the accumulation of abscisic acid (ABA) and ROS (Hedrich and Shabala 2018). Under drought conditions, ABA production increases in roots and is translocated to shoots via the xylem, triggering stomatal closure to reduce transpiration loss (Jacobsen et al. 2009). While this regulation preserves water status, it imposes diffusive limitations on photosynthesis by restricting CO_2_ uptake (Killi and Haworth 2017). However, quinoa maintains photosynthetic capacity even under severe water deficits, suggesting robust non-stomatal resilience mechanisms (Killi and Haworth 2017). Furthermore, agronomic strategies such as alternate root-zone drying have been shown to enhance water use efficiency (WUE), possibly by optimizing this ABA signaling mechanism, allowing the plant to maintain water status while partially closing stomata (Yang et al. 2016).

Root architecture plays a pivotal role in plants’ ability to access soil moisture. Quinoa typically develops a deep, dense root system that facilitates water uptake from deeper soil layers. The plasticity of this system is evident when comparing genotypes from different habitats. Genotypes from arid environments exhibit faster taproot elongation and produce longer, coarser, and more numerous root segments compared to those from humid environments (Alvarez-Flores et al. 2018). This phenotypic flexibility allows the plant to forage effectively for water during moisture deficits. Additionally, soil amendments such as biochar have been found to significantly increase taproot biomass and overall plant growth under drought conditions, further improving WUE and drought tolerance (Kammann et al. 2011).

ROS has dual roles in plant abiotic stress response. Low levels of ROS function as a secondary messenger in stress signaling, while high levels of ROS cause oxidative damage to the cellular machinery. Although the function of ROS in quinoa stress signaling remains to be studied, quinoa employs sophisticated biochemical defense systems to mitigate oxidative damage caused by abiotic stress, particularly drought and temperature stress. Both heat and drought stress trigger the accumulation of reactive oxygen species (ROS), such as hydrogen peroxide (H_2_O_2_), which can be toxic to cellular components (Hinojosa et al. 2019b). To counteract this, plants upregulate the activity of antioxidant enzymes, including superoxide dismutase (SOD), peroxidase (POD), and catalase (CAT). For instance, under combined drought and heat stress, tolerant genotypes of quinoa have been observed to better increase antioxidant enzyme activities to detoxify ROS and maintain cellular homeostasis (Abbas et al. 2023; Hinojosa et al. 2019b). Non-enzymatic antioxidants, including flavonoids, tocopherols, and betalains, likely also play a role in scavenging free radicals. Interestingly, the abundance of peroxisomes, which contain ROS scavenging enzymes, was shown to be increased by heat, drought, or the combination of both, and is a good predictor of yield loss (Hinojosa et al. 2019b).

Quinoa exhibits significant frost tolerance, capable of surviving temperatures as low as −8 °C depending on the phenological stage, though the flowering stage remains the most sensitive (Jacobsen et al. 2005). The mechanism for frost resistance primarily involves the avoidance of ice formation within tissues. This is also achieved through the accumulation of soluble sugars (particularly sucrose) and proline, which act as cryoprotectants and stabilize cellular membranes (Jacobsen et al. 2007; Rosa et al. 2009). Furthermore, biochemical analyses of seedlings have shown that low temperatures induce specific changes in sucrose-starch partitioning and related enzyme activities, facilitating osmotic adjustment (Rosa et al. 2009). At the molecular level, the accumulation of dehydrin proteins in seeds and embryos has been identified as a protective mechanism against desiccation and low-temperature stress.

### Key limitations and stress sensitivity

While quinoa is renowned for its resilience to abiotic stresses such as drought and salinity, its expansion into diverse global environments faces significant constraints. Current research highlights specific sensitivities to high temperatures, preharvest sprouting, and soil waterlogging, each of which can severely compromise yield and grain quality.

#### High temperature

Elevated temperatures, particularly during the reproductive stages, pose a major barrier to quinoa cultivation in lower latitudes and regions prone to heatwaves (Hinojosa et al. 2018). Most grain crops are highly sensitive to heat stress during the reproductive stage, and this is also the case for quinoa (Tovar et al. 2020). In most genotypes, heat stress compromises the drought and salt stress tolerance potential, negatively affecting growth, yield, and the internal K^+^/Na^+^ ratio (Abbas et al. 2023). Differential heating experiments reveal that shoot sensitivity is the primary driver of yield loss, whereas root heating has a negligible effect on agronomic parameters (Tovar et al. 2020). Increased night temperatures also negatively affect grain yield, biomass, and grain number (Lesjak and Calderini 2017). Temperatures exceeding 32–35 °C can lead to substantial yield losses, with studies showing reductions of 31–85% depending on the genotype and the specific timing of the stress (Alvar-Beltrán et al. 2020; Lesjak and Calderini 2017; Tovar et al. 2020). Heat stress primarily impacts the reproductive organs; while pollen morphology generally remains intact, high temperatures can reduce pollen viability by 30% to 80% (Hinojosa et al. 2019a; Xu et al. 2025). The most heat-sensitive period for pollen viability appears to be 8–10 days prior to flowering (Xu et al. 2025). Furthermore, heat stress limits pollination by causing flowers to remain closed during the day (Tovar et al. 2020).

Beyond yield, heat stress alters the nutritional profile of the seeds. Compositional changes also include increased protein and fiber content, alongside decreased contents of fats and carbohydrates (Matías et al. 2021). Interestingly, high temperatures during anthesis can significantly modify the concentration of minerals such as calcium, iron, and zinc in the seeds, with effects persisting even after the stress is removed (Tovar et al. 2022).

Most quinoa genotypes increase stomatal conductance and photosynthetic rates under high heat to facilitate transpirational cooling, provided water is available (Eustis et al. 2020; Hinojosa et al. 2019b). However, this comes at the cost of lower intrinsic water use efficiency (Eustis et al. 2020). When heat and drought stress co-occur, quinoa exhibits decreased stomatal closure and transpiration, and the highest level of ROS compared to any single stress (Hinojosa et al. 2019b).

Transcriptomic analyses indicate a downregulation of photosynthesis-related genes under long-term heat stress and identified the upregulation of heat shock factors (HSFs), such as *CqHsfs3* and *CqHsfs9*, which regulate downstream heat shock proteins (HSPs) to confer thermotolerance (Xie et al. 2023). Additionally, both transcriptome and metabolome data suggest that regulation of purine metabolism and the heat shock response are closely linked, with tolerant genotypes showing distinct regulation of adenine and guanine recycling pathways (Xie et al. 2023).

Exogenous application of bio-regulators, such as myo-inositol, has shown promise in mitigating oxidative damage and protecting photosynthetic efficiency during thermal stress (Alshammari et al. 2025). In addition, wild relatives of quinoa, particularly *Chenopodium berlandieri*, have demonstrated superior yield stability and pollen viability under extreme heat compared to cultivated quinoa, making them valuable resources for introgression breeding (Xu et al. 2025).

#### Preharvest sprouting

Preharvest sprouting (PHS), the germination of seeds on the maternal plant prior to harvest, is a trait closely related to seed dormancy. The phenomenon is driven by the lack of strong seed dormancy in many modern quinoa cultivars, a trait that was likely selected against during domestication to ensure uniform stand establishment (McGinty et al. 2021). It was observed that quinoa varieties with thinner seed coats have lower dormancy and higher susceptibility to PHS, whereas darker, thicker seed coats are associated with stronger dormancy (McGinty et al. 2021). Environmental factors also play a role; high temperatures and long photoperiods during seed development can enhance secondary dormancy (McGinty et al. 2021). Quinoa varieties with strong seed dormancy have been reported and used for generating progenies with increased PHS resistance through hybridization (Ceccato et al. 2015). The genetic basis and molecular mechanisms of PHS in quinoa remain to be fully determined.

Early studies in Arabidopsis and cereals have identified tens of genes regulating seed dormancy and PHS (vivipary), most of which have orthologs in quinoa (Gubler et al. 2005; Tai et al. 2021). Dormancy is regulated by the balance between abscisic acid (ABA), which promotes dormancy, and gibberellins (GA), which promote germination (release of dormancy). Given that ABA- and GA-related components are conserved in quinoa (Zou et al. 2017), they represent promising targets for molecular breeding. Although dormancy benefits PHS prevention, excessive dormancy could result in asynchronous germination, which is another unfavorable trait in agricultural practice (Nonogaki and Nonogaki 2017). Thus, the molecular mechanisms of GA and ABA biosynthesis or signaling components during quinoa seed development and germination need to be further studied. In addition, the use of plant growth regulators, such as Paclobutrazol (a GA inhibitor) (Gómez et al. 2011) or exogenous ABA (Cao et al. 2017) applications prior to harvest, can transiently induce dormancy or inhibit germination, offering a potential management tool for reducing PHS risk.

#### Flooding and waterlogging

While quinoa is drought-tolerant, it is highly sensitive to excess soil water content. Waterlogging causes hypoxia in the root zone, leading to severe growth inhibition and yield penalties (Sasidharan et al. 2018). Waterlogging significantly reduces plant height, leaf area, and root biomass (Guo et al. 2024; Nguyen et al. 2024). The severity of the damage is highly dependent on the developmental stage. In the greenhouse, quinoa is most vulnerable during anthesis, where waterlogging can reduce yields by over 70% due to grain abortion and reduced harvest index (Vásquez et al. 2025). Comparative studies indicate that waterlogging is more detrimental to quinoa growth than drought or salinity stress (Loc Nguyen et al. 2025).

Quinoa responds to flooding through morphological and metabolic adaptations. Morphologically, tolerant genotypes may form adventitious roots to facilitate oxygen uptake (Guo et al. 2024). Metabolically, roots shift from aerobic respiration to fermentation processes, accumulating soluble sugars (sucrose, glucose) to maintain energy levels (Guo et al. 2024). A key response mechanism involves the accumulation of γ-aminobutyric acid (GABA), which helps regulate intracellular pH and acts as a signaling molecule to activate antioxidant systems (Guo et al. 2024).

Transcriptomic analyses reveal that waterlogging triggers the expression of stress-responsive transcription factors, particularly from the AP2/ERF, MYB, and WRKY families (Wang et al. 2025b). These regulators modulate the biosynthesis of secondary metabolites, such as flavonoids and phenolic acids, which enhance antioxidant capacity and scavenge reactive oxygen species generated by hypoxic stress (Guo et al. 2024; Jiang et al. 2025; Wang et al. 2024). Furthermore, pathways involving linoleic acid metabolism are activated to maintain cell membrane integrity under stress (Guo et al. 2024).

Improvements in waterlogging tolerance rely on identifying and breeding genotypes with superior recovery capacity and metabolic adaptability. Genotypes such as ‘Dianli-188’ and ‘G18’ have exhibited high tolerance indices and stability across test environments (Guo et al. 2024; Nguyen et al. 2024). However, much larger screens are needed to identify varieties that perform better under more waterlogged conditions. Additionally, agronomic management to avoid waterlogging during the critical anthesis stage is essential for preserving yield.

## Genomic resources and genetic tools

### Genomic resources and structure

Quinoa has an allotetraploid genome (2n = 4x = 36, AABB) with a high content of repetitive sequences. Early efforts to sequence the genome utilized hybrid sequencing approaches on inbred lines to manage heterozygosity. For instance, the draft genome of the inbred accession ‘Kd’ was assembled using Illumina and PacBio technologies, yielding a 1.0 Gbp assembly that facilitated the creation of the Quinoa Genome DataBase (Yasui et al. 2016). Subsequently, a high-quality chromosome-scale reference genome for the coastal Chilean accession PI 614886 (QQ74) was produced using single-molecule real-time sequencing, optical mapping, and chromosome-contact maps. This assembly spanned 1.39 Gb, with 90% of the genome contained in 439 scaffolds (Jarvis et al. 2017). Concurrently, a reference genome for the ‘Real’ cultivar was generated, covering 1.34 Gb and identifying 54,438 protein-coding genes (Zou et al. 2017).

These reference assemblies have proved critical for understanding the molecular basis of key agronomic traits. The QQ74 reference genome facilitated the identification of TSARL1, a transcription factor regulating the production of anti-nutritional triterpenoid saponins in seeds (Jarvis et al. 2017). By characterizing a mutation in *TSARL1* that causes alternative splicing and a premature stop codon, researchers identified a genetic marker for breeding “sweet” (saponin-free) quinoa varieties (Jarvis et al. 2017). Additionally, the genome has enabled the study of salinity resistance mechanisms. Genome analysis of the ‘Real’ cultivar highlighted the expansion of gene families involved in ion transport and ABA homeostasis, correlating these expansions with quinoa’s stress tolerance and high nutritional value, particularly its lysine-rich seed storage proteins (Zou et al. 2017).

A recent improvement to the PI 614886 reference, termed QQ74-V2, utilized in vivo Hi-C data to produce a highly contiguous assembly where 90.5% of the sequence is contained in 18 chromosome-scale scaffolds (Rey et al. 2023). This improved assembly revealed significant structural variations, including a large 52 Mb pericentromeric inversion on chromosome 3B that distinguishes coastal from highland ecotypes (Rey et al. 2023).

To capture the full genetic diversity of the genus and utilize wild relatives for crop improvement, research is expanding toward a Chenopodium pangenome. Recent analyses of 12 Chenopodium species, encompassing the eight known genome types (A–H), have revealed substantial variation in genome size and structure (Jaggi et al. 2025; Ludwig et al. 2025; Mangelson et al. 2019; Rey et al. 2023; Young et al. 2023). The genomes range from approximately 370 Mb in the D-genome diploid *C. acuminatum* to over 1.6 Gb in hexaploid species.

Comparative pangenomic studies have highlighted the role of repetitive elements in genome evolution. The expansion of long terminal repeat (LTR) retrotransposons, particularly *Gypsy* elements, has been identified as a major driver of genome size differences (Jaggi et al. 2025). For instance, in the hexaploid *C. formosanum* (BCD genome), the B subgenome has significantly expanded due to the accumulation of *Gypsy* elements compared to the C and D subgenomes (Jaggi et al. 2025). Similarly, different families of satellite DNAs show differential amplification across diploid and polyploid lineages, with the f5 family being specifically amplified in the B subgenome (Jaggi et al. 2025). This differential repeat accumulation supports the hypothesis that the B subgenome is more dynamic than the A subgenome in polyploid lineages.

The pangenome also clarifies evolutionary relationships and hybridization events. Orthogroup phylogenies have confirmed the eight major genomic lineages and resolved the placement of the F genome as a sister group to the D genome clade. Furthermore, the sequencing of *C. album* confirmed its allohexaploid status (2n = 6x = 54) and provided resources for understanding this globally distributed weed.

Pangenome analysis has identified a vast repertoire of “shell” gene families that are not present in the core genome. Approximately 65% of gene families in the genus were classified as shell genes (Jaggi et al. 2025). These variable gene pools are enriched for biological processes involved in environmental adaptation, such as defense responses and metabolic pathways. For example, lineage-specific expansions of *FLOWERING LOCUS T-LIKE* (*FTL*) genes, particularly in clade II, suggest dynamic evolution related to niche adaptation (Jaggi et al. 2025). The characterization of these diverse genomic resources provides a foundation for identifying beneficial alleles in wild taxa for introgression into domesticated quinoa, potentially enhancing traits such as disease resistance and climate resilience.

### Population structure and genetic mapping

The genetic diversity of quinoa (*Chenopodium quinoa* Willd.) is shaped by its evolutionary history and domestication in the Andes. Quinoa is traditionally classified into five ecotypes based on the cultivation area, including Sea level, Yungas, Inter-Andean valley, Highlands, and Salt Flatlands (Rojas and Pinto 2015). Population analyses based on genome resequencing data consistently classify quinoa germplasm into two primary distinct pools: the Andean Highland type and the Chilean Coastal (Lowland) type (Fondevilla et al. 2024; Mizuno et al. 2020; Patiranage et al. 2022; Zhang et al. 2017). While Principal Component Analysis (PCA) and phylogenetic trees often resolve the population into these two main clusters, finer substructures exist. For instance, the Andean Highland group is frequently subdivided into Northern (Peru) and Southern (Bolivia) subgroups. Recent studies utilizing high-density SNP markers on large diversity panels (201 to 310 accessions) have confirmed this clustering, identifying three major groups: Lowland/Coastal accessions and two distinct Highland groups representing Peruvian and Bolivian germplasm (Mizuno et al. 2020; Rahman et al. 2024).

Genetic differentiation between these populations is significant. An F_ST_ value of 0.36 was reported between Highland and Lowland populations, indicating strong differentiation (Patiranage et al. 2022). Genetic diversity analysis reveals that diversity tends to be higher in the Southern Highland group compared to the Northern Highland group, with Chilean Coastal germplasm showing distinct genetic profiles (Zhang et al. 2017). Furthermore, linkage disequilibrium (LD) decay varies significantly by population. LD decay was found to be rapid in Highland populations (6.5 kb at *r*^*2*^ = 0.2) compared to Lowland populations (49.8 kb), suggesting different breeding histories and selection pressures (Patiranage et al. 2022). However, when analyzing broader germplasm sets, average genome-wide LD decay has been estimated at approximately 118.5 kb (Rahman et al. 2024).

Genome-wide association studies (GWAS) have become a critical tool for dissecting the genetic architecture of agronomic traits in crops, utilizing the high density of single nucleotide polymorphisms (SNPs) available from re-sequencing data (Liu and Yan 2019). Recent studies in quinoa have employed large diversity panels genotyped with millions of SNPs to map traits such as flowering time, seed weight, and disease resistance (Fondevilla et al. 2024; Habib et al. 2024; Patiranage et al. 2022; Rahman et al. 2024). For example, a GWAS of 303 accessions identified 600 consistent marker-trait associations (MTAs) across multiple years (Patiranage et al. 2022).

However, the application of GWAS in quinoa presents specific challenges. The strong population structure between Highland and Lowland ecotypes can lead to false positives or mask true associations (Nordborg and Weigel 2008). In analyses of salt tolerance, Mixed Linear Models (MLM), which account for both population structure and kinship, identified significantly fewer associations than General Linear Models (GLM), suggesting that strong differentiation among populations may limit the power of GWAS for certain traits (Mizuno et al. 2020). To overcome limitations in standard univariate GWAS, alternative approaches such as machine learning have been tested. For multiclass traits like seed color, an extreme gradient boosting (XGBoost) model outperformed classical GWAS, achieving 88% prediction accuracy compared to linear mixed models, which struggled to identify useful variance for multiclass phenotypes (Sandell et al. 2024). Additionally, multivariate GWAS approaches analyzing cross-phenotypes (e.g., combining days to flowering, maturity, and plant height) have successfully identified pleiotropic loci that might be missed by single-trait analyses (Patiranage et al. 2022).

### Functional genomics tools

Recent advances have established virus-mediated transient expression systems and novel tissue culture-based transformation protocols as viable tools for analyzing gene function in quinoa. Virus-induced gene silencing (VIGS) is a post-transcriptional gene silencing technique used to transiently knock down the expression of endogenous genes (Dommes et al. 2019). In quinoa, the Apple latent spherical virus (ALSV) vector system has emerged as a robust tool for functional genomics, serving as the primary method currently available for endogenous gene function analysis (Ogata et al. 2021). ALSV is an RNA virus that can systemically infect a variety of crops, including quinoa, and acts as a silencing vector without inducing severe disease symptoms (Kasajima et al. 2017). The principle relies on the plant’s antiviral defense mechanism. When the ALSV vector carrying a fragment of a target quinoa cDNA replicates within the host, it generates double-stranded RNA intermediates. These are processed by the host’s Dicer-like proteins into small interfering RNAs (siRNAs), which guide the Argonaute complex to degrade the complementary endogenous mRNA of the target gene, resulting in specific gene knockdown (Dommes et al. 2019).

The ALSV-VIGS system has been successfully applied across diverse quinoa genotypes, including Northern highland, Southern highland, and Lowland sub-populations (Ogata et al. 2021). This tool has been utilized to rapidly dissect gene functions related to pigment biosynthesis, plant architecture, and stress resistance in quinoa. For instance, VIGS confirmed the roles of *CqDODA1* and *CqCYP76AD1* in betalain biosynthesis, and identified the *CqRHT1* homolog as a negative regulator of gibberellin signaling through its overgrowth phenotype (Ogata et al. 2021). VIGS validated the function of genes like *REBC* (*Reduced number of EBCs*) and *REBC-LIKE1* in EBC development (Kobayashi and Fujita 2024) and confirmed the functions of sodium transporters CqHKT1;1, CqHKT1;2, and CqSOS1 in excluding Na^+^ from leaves under saline conditions (Kobayashi et al. 2025).

Beyond gene silencing, ALSV has also been utilized for virus-mediated overexpression (VOX), successfully expressing Green Fluorescent Protein (GFP) in quinoa roots (Ogata et al. 2021). While typically transient, ALSV-VIGS was reported to occasionally be transmitted to progeny through seeds, albeit at a low frequency (less than 5%) (Ogata et al. 2021).

Establishment of efficient tissue culture and genetic transformation protocols is essential for stable genetic engineering and genome editing. While quinoa has traditionally been recalcitrant to in vitro regeneration, significant progress has been made in optimizing culture conditions and developing stable transformation methods (Porras-Murillo et al. 2025; Sidorov et al. 2024).

Successful tissue culture requires sterile establishment, typically achieved using sodium hypochlorite and ethanol surface sterilization of seeds. In vitro germination is highly efficient on Murashige & Skoog (MS) medium under standard photoperiods. Regeneration pathways in quinoa include direct organogenesis (shoot formation from meristems), indirect organogenesis (shoots from callus), and somatic embryogenesis (Porras-Murillo et al. 2025).

A major bottleneck in quinoa tissue culture has been premature senescence of shoots and low multiplication rates. Recent progress addressed this issue through optimizing the growth medium. A recent study shows that using triple-strength MS (3MS) macronutrients combined with specific growth regulators (BA and NAA) is effective in overcoming senescence, yielding robust, elongated shoots (Xie et al. 2025). Furthermore, a highly effective and stable genetic transformation system has been developed using *Agrobacterium tumefaciens*-mediated floral culture (Sidorov et al. 2024). This method utilizes highly meristematic chopped inflorescences as explants, allowing for the regeneration of non-chimeric transgenic shoots that can self-pollinate in vitro or be grafted to produce viable transgenic seeds. Other methods, such as *Agrobacterium rhizogenes*-mediated transient expression in hairy roots (Imamura et al. 2018) and *Agrobacterium tumefaciens*-mediated transient transformation for rapid testing of CRISPR constructs (Xiao et al. 2022), further support functional genomics studies in quinoa.

Collectively, these tools – ranging from transient VIGS for rapid gene function assays to stable transformation via floral cultures – now permit sophisticated genetic studies in quinoa, facilitating the investigation of agronomic traits and stress-related molecular mechanisms.

## Future perspectives and strategies

### Sustainable cultivation on marginal lands

The global expansion of quinoa cultivation is increasingly driven by the imperative to utilize marginal lands – areas characterized by soil salinity, water scarcity, and extreme temperatures – to ensure food security without competing for prime arable land (Choukr-Allah et al. 2016). Quinoa’s status as a facultative halophyte makes it a strategic crop for the rehabilitation of salt-affected soils, capable of completing its life cycle in salinity levels up to 400 mM NaCl, conditions where major staple crops fail. To achieve these goals, researchers are advocating for the domestication of naturally stress-resistant plants (NSRPs) by leveraging the genetic architecture of wild relatives or semi-domesticated crops and using modern genomic tools to introgress high-yield and good-quality traits into stress-resistant backgrounds (Zhang et al. 2018).

To facilitate sustainable expansion into these environments, integrated agronomic strategies must be adopted. For example, the implementation of deficit irrigation strategies, particularly during the vegetative stages, has been shown to stabilize yields and maximize crop water productivity in arid regions such as the Middle East and North Africa (Choukr-Allah et al. 2016). Seed germination remains a relatively sensitive stage to soil salinity in the quinoa cultivation cycle. A promising frontier involves the inoculation of quinoa with beneficial microorganisms. Plant growth-promoting rhizobacteria (PGPR) and arbuscular mycorrhizal fungi (AMF) can enhance nutrient uptake and activate antioxidant defense mechanisms, thereby alleviating ionic and osmotic stress (Kumar et al. 2015; Pitzschke 2018; Singh et al. 2022).

Breeding objectives must prioritize yield stability and adaptation to these non-native, stressful environments over maximum yield potential alone. A major constraint for global expansion is the sensitivity of quinoa to high temperatures and photoperiod regimes different from its Andean origins (Lopez-Marques et al. 2020). Additionally, the development of varieties resistant to pre-harvest sprouting is critical for expanding cultivation to regions with wet harvest seasons and irregular rainfalls caused by global climate change(McGinty et al. 2021). This is particularly important as rainfall becomes more irregular due to global climate change; even traditionally arid areas could experience extended rainfall during harvest season (Hirabayashi et al. 2013).

### Challenges in germplasm standardization and communication

The rapid globalization of quinoa since 2013 has outpaced the development of standardized seed systems and international regulatory frameworks, creating challenges in germplasm exchange and classification. The restrictive nature of international agreements, such as the Nagoya Protocol and the absence of quinoa in the Multilateral System of the International Treaty on Plant Genetic Resources for Food and Agriculture, hampers the cross-border exchange of genetic materials necessary for adapting the crop to new latitudes (Alandia et al. 2020). Another technical difficulty of germplasm exchange lies in the complexity of mixed germplasms; Andean farmers traditionally maintain high levels of genetic diversity within their fields to ensure resilience against variable climates (Fuentes et al. 2012). While this biodiversity is an asset for subsistence farming, the lack of genetic uniformity complicates industrial standardization and the distinctness, uniformity, and stability (DUS) required for variety registration in international markets. Establishing a Global Collaborative Network on Quinoa (GCN-Quinoa) has been proposed to facilitate the standardization of descriptors and promote the sharing of pre-breeding genetic material, ensuring that researchers worldwide can effectively communicate and integrate data regarding agronomic performance and stress tolerance (Murphy et al. 2016). Finally, the standardization of phenotyping protocols and terminology is also needed among the global quinoa community to reduce inconsistent reporting of experimental conditions (such as light intensity, soil composition, and specific growth stages) and increase the reproducibility of studies and the ability to conduct meta-analyses.

### Integrating modern technologies for accelerated breeding

Domestication of the primary crops took thousands of years, while genetic analyses indicate that homologous genes or similar signaling pathways were altered during the domestication of similar traits in different crops (Lenser and Theissen 2013). New generations of breeding technologies should be employed to speed up the pace (Farooq et al. 2024; Hua et al. 2019; Xu et al. 2022). Phenomics, utilizing high-throughput phenotyping platforms, allows for the non-destructive, simultaneous measurement of numerous morphological and physiological traits across large populations. Technologies such as Unmanned Aerial Vehicles (UAVs) equipped with thermal and hyperspectral imaging sensors are becoming critical for dissecting complex traits like salinity and drought tolerance in the field, enabling the capture of dynamic responses that single-point measurements miss. This accumulation of phenotypic data is vital for bridging the genotype-to-phenotype gap.

Complementing phenomics, the application of predictive modeling and Artificial Intelligence (AI) is revolutionizing genomic selection (GS) (Xu et al. 2020). GS utilizes genome-wide markers to predict the breeding value of individuals, allowing for the selection of superior genotypes without the need for extensive phenotypic evaluation in every generation. This approach is particularly effective for complex, polygenic traits such as yield and abiotic stress tolerance, where traditional marker-assisted selection often falls short. Furthermore, the availability of the quinoa reference genome will facilitate the use of precise gene-editing tools, such as CRISPR/Cas9, to functionally validate candidate genes and introduce targeted mutations for traits like saponin reduction and heat tolerance. “Transgene-free” editing strategies, such as the viral delivery of genome editors (Weiss et al. 2025; Yoshida et al. 2024), offer a viable pathway to bypass regulatory hurdles in some regions and accelerate the development of climate-resilient quinoa varieties. Combined with “speed breeding” techniques that shorten generation times (Chiurugwi et al. 2018), these technological integrations promise to rapidly deliver locally adapted quinoa cultivars to meet global food security demands.

## Conclusions

Quinoa’s combination of exceptional nutritional quality and profound abiotic stress resilience makes it a cornerstone crop for sustainable agriculture in an era defined by climate change and mounting salinization. The research reviewed here demonstrates significant advances across all levels of biological inquiry: from deepening understanding of the physiological mechanisms of salt management (including the ongoing debate over the role of EBC) to the molecular dissection of its allotetraploid genome. The availability of multiple high-quality reference genomes and the development of the Chenopodium pangenome have laid the foundation for accelerated functional genomics, enabling the molecular identification of key traits. Furthermore, the reported molecular tools, including the ALSV-based VIGS system and stable Agrobacterium-mediated floral transformation, now provide the necessary tools for genetic engineering of the quinoa genome. However, the path to full global exploitation of quinoa also faces challenges. Critical biological limitations – specifically sensitivity to high temperatures, waterlogging, and preharvest sprouting – must be overcome through targeted breeding efforts. Concurrently, the community must address challenges in germplasm standardization, regulatory frameworks, and consistent phenotyping protocols to ensure that genetic material can be effectively shared and deployed worldwide. By fully integrating high-throughput phenomics and AI-driven Genomic Selection with precise genome editing, the timelines for breeding locally adapted, high-yielding, and climate-resilient quinoa cultivars can be dramatically shortened. Quinoa is not merely a promising pseudo-cereal; it is a vital model system for translating halophytic mechanisms into resilient cropping systems, ensuring both nutritional security and the sustainable utilization of marginal agricultural lands.

## Data Availability

Data request should be addressed to H.Z. (zhangheng82@sjtu.edu.cn).

## Abbreviations

GS: Genomic Selection
FAO: Food and Agriculture Organization of the United Nations
MENA: Middle East and North Africa
EBC: Epidermal Bladder Cell
Na^+^: Sodium ion
K^+^: Potassium ion
NHX: Sodium hydrogen exchanger
ROS: Reactive oxygen species
ABA: Abscisic acid
WUE: Water use efficiency
H_2_O_2_: Hydrogen peroxide
SOD: Superoxide dismutase
POD: Peroxidase
CAT: Catalase
HSF: Heat shock factor
HSP: Heat shock protein
PHS: Preharvest sprouting
GA: Gibberellin
GABA: γ-Aminobutyric acid
LTR: Long terminal repeat
*FTL*: *FLOWERING LOCUS T-LIKE*
PCA: Principal Component Analysis
LD: Linkage disequilibrium
GWAS: Genome-wide association studies
SNP: Single nucleotide polymorphism
MTA: Marker-trait association
MLM: Mixed Linear Model
GLM: General Linear Model
XGBoost: Extreme gradient boosting
VIGS: Virus-induced gene silencing
ALSV: Apple latent spherical virus
siRNA: Small interfering RNA
VOX: Virus-mediated overexpression
GFP: Green Fluorescent Protein
MS: Murashige & Skoog
DUS: Distinctness, uniformity, and stability
GCN-Quinoa: Global Collaborative Network on Quinoa
UAV: Unmanned Aerial Vehicle
BA: 6-benzyl adenine
NAA: 1-naphthaleneacetic acid
